# Vaginal Laser Treatment for the Genitourinary Syndrome of Menopause in Breast Cancer Survivors: A Narrative Review

**DOI:** 10.7759/cureus.45495

**Published:** 2023-09-18

**Authors:** Nobuo Okui

**Affiliations:** 1 Dentistry, Kanagawa Dental University, Yokosuka, JPN

**Keywords:** breast cancer survivors, fractional microablative co2 vaginal laser, non-ablative erbium:yag laser, genitourinary syndrome of menopause, vulvovaginal atrophy

## Abstract

Vulvovaginal atrophy (VVA) is a chronic condition resulting from reduced estrogen levels during menopause. The North American Menopause Society and the International Society for the Study of Women's Sexual Health suggested the term "genitourinary syndrome of menopause" (GSM) to indicate the broader aspects of VVA. Breast cancer treatments, such as chemotherapy and endocrine therapy, can induce early and abrupt menopausal symptoms, including GSM, which negatively affects sexual function and the quality of life of the survivors. Vaginal laser therapy has emerged as a safe and effective option for the management of GSM in breast cancer survivors (BCSs). Two main types of lasers, the non-ablative erbium:YAG laser and fractional microablative CO_2_ vaginal laser, have been evaluated for GSM treatment. While there are few randomized controlled trials (RCTs) on the subject of BCSs, a wealth of prospective and retrospective studies have highlighted the beneficial effects of vaginal laser therapy on the symptoms of VVA, vaginal health, sexual function, and overall quality of life. More comprehensive research is essential to confirm its enduring effectiveness and safety, with a focus on conducting standardized and meticulously controlled investigations. This study is a narrative review that summarizes clinical trials ranging from the earliest to the most recent ones on laser treatment for GSM in BCSs.

## Introduction and background

Breast cancer is a major concern and the most frequently identified cancer globally [1]. Adjuvant therapies for breast cancer, such as hormonal treatment and chemotherapy, can lead to chronic and progressive conditions related to decreased estrogen levels; specifically, they can induce vulvovaginal atrophy (VVA), which is more common in the postmenopausal phase with a prevalence of approximately 50%. Breast cancer treatment, including surgery, endocrine therapy, and chemotherapy, may cause or exacerbate VVA, which affects the genital and lower urinary tracts [2-5]. The International Society for the Study of Women's Sexual Health and the North American Menopause Society adopted the term "genitourinary syndrome of menopause" (GSM) as a more comprehensive and accurate name than VVA [2-5]. GSM is characterized by a series of genital, urinary, and sexual symptoms in post-menopausal women caused by reduced estrogen stimulation; GSM generally presents with symptoms, such as vaginal dryness, itching, burning, dyspareunia, and urinary disturbances [2-5].

GSM tends to be a chronic condition that does not improve over time. Therefore, finding safe and effective treatment options for GSM is crucial, particularly in women with a history of breast cancer [2,5]. Currently, vaginal laser therapy has shown promising results in improving symptoms in postmenopausal women, comparable to those of local estrogen therapy; therefore, determining its potential benefits in breast cancer survivors (BCSs) has attracted much attention [6-8]. Given the information above, conducting a narrative review encompassing laser therapy for GSM in BCSs is valuable, from early trials to recent research.

## Review

Diagnosing and evaluating GSM

VVA and GSM impact postmenopausal women substantially, but many symptomatic women are unaware of these conditions as medical issues. Diagnosis requires a comprehensive evaluation of medical history and gynecological examination. Proactive inquiry about suggestive symptoms is essential for identifying GSM [9].

Several physical characteristics might be observed in GSM, including a thinning or absence of pubic hair, a reduction in the size of the mons pubis, and potential fusion of the labia minora. The vaginal interior may become narrower and have a dry, delicate, pale vaginal tissue, sometimes exhibiting a glossy appearance. Furthermore, the vaginal elasticity decreases, the cervix may shorten, and redness or pinpoint bleeding from the vagina might be observed. The dominant bacterial population in the vagina may change, leading to abnormal vaginal discharge. Regarding urinary findings, urethral atrophy and redness might be accompanied by urinary symptoms. The vaginal acidity in GSM is usually elevated (pH > 5.0) compared with that in a healthy vagina, which typically has a pH of approximately 3.8-4.5 [2].

The frequently utilized research indices to evaluate the effectiveness of GSM treatment include the Visual Analogue Scale (VAS), Vaginal Health Index Score (VHIS), Vaginal Maturation Index (VMI), Female Sexual Function Index (FSFI), and FSFI for breast cancer patients (FSFI-BC) [10]. The VAS subjectively measures the symptom intensity. The FSFI assesses female sexual function through six domains, providing scores ranging from 2 to 36 (higher scores indicate better sexual function) [11]. The FSFI-BC is an adapted version of the FSFI, specifically designed for patients with breast cancer, which incorporates two additional subscales to assess changes related to breast cancer and post-pain experiences [11]. The VHIS determines the degree of vaginal atrophy by evaluating five parameters: vaginal elasticity, pH, epithelial mucosa, secretions, and tissue hydration. The total score ranges from 5 to 25 (with reduced scores indicating more pronounced atrophy) [12].

A debate surrounds the VHIS examination method owing to its exclusive focus on vaginal pH measurement. By contrast, the VMI utilizes the ratio of three vaginal epithelial cell types (superficial, intermediate, and parabasal), rendering it an objective tool. Vaginal atrophy is believed to occur under conditions of low estrogen levels, resulting in increased parabasal cells. Nevertheless, the VMI is seldom utilized in clinical trials associated with GSM [10,13]. To comprehensively assess GSM, clinical and research settings can benefit from employing a combination of subjective and objective evaluation tools [14].

Impact of breast cancer treatment

Breast cancer treatment involves a comprehensive approach that combines multiple therapies. Combination therapy enhances treatment effectiveness and efficiency. Neoadjuvant therapy is employed in cases of locally advanced or inoperable tumors to reduce their size and render them surgically manageable [15]. Chemotherapy can negatively impact ovarian function by causing toxicity to immature oocytes and the surrounding granulosa cells, ultimately destroying ovarian follicles. Consequently, this can cause treatment-induced ovarian function suppression [16]. Cancer treatments, including chemotherapy and radiation therapy, are believed to impact the ovarian function of young women and girls significantly. Major considerations include adverse health effects and the loss of fertility. These effects are suggested to contribute to the development of GSM [17].

A majority of patients with breast cancer have hormone receptor-positive tumors; overexpression of estrogen receptors (ER) and/or progesterone receptors (PR) is observed in up to 77% of patients diagnosed with breast cancer [18]. Information about ER and PR is vital for patient management, and the semiquantitative assessment of ER and PR is essential for prognosis and treatment [19]. Hormone therapy for women with ER-positive breast cancer commonly involves maintaining it as a standard treatment for five to 10 years [20]. For breast cancer patients, tamoxifen or gonadotropin-releasing hormone analogs are selected, while in postmenopausal women, tamoxifen and aromatase inhibitors (AIs) are indicated [20]. These medications can potentially cause atrophy of the genitourinary tract in women with abundant ER [21]. Different types of endocrine therapies have diverse impacts on the vaginal canal, leading to variations in the frequency and severity of side effects.

AIs inhibit the enzyme involved in estrogen synthesis from androgens [22]; these medications decrease estrogen's physiological concentrations [23]. Furthermore, AIs may increase the severity of GSM symptoms by locally inhibiting aromatase in vaginal tissues. It is suggested that decreasing the proliferation of vaginal epithelium may be associated with the severity of vaginal atrophy scores, vaginal pH, and reduced sexual function [24-27].

Tamoxifen is a selective ER modulator; its mechanism of action involves binding to ER in both normal breast and breast cancer cells, competing with estrogen, and exerting anti-tumor effects [28]. Tamoxifen blocks circulating estrogen [25], suppressing estrogen effects in tissues other than the breast. Considering that estrogen can potentially improve vaginal dryness [29], GSM symptoms in patients undergoing tamoxifen therapy are reported to have a high incidence rate [30].

Prevalence of GSM in BCSs

Up to 84% of postmenopausal women generally experience genitourinary symptoms [2]. Among BCSs, the prevalence of GSM symptoms is even higher [31]. Cross-sectional studies have reported that 35-91% of BCSs experience at least one GSM symptom. Among them, vasomotor symptoms, sexual problems, mood fluctuations, and sleep disturbances are frequently reported in 74% of cases [26,32,33]. Vaginal dryness and dyspareunia are commonly reported symptoms among BCSs [31-35].

Sexual difficulty and vaginal dryness can be observed in many postmenopausal BCSs compared to those in premenopausal BCSs (38.7% vs. 15.9% and 61.5% vs. 23.4%, respectively) [36]. Differences in treatment approaches are also observed in relation to endocrine therapy, where BCSs receiving AIs report GSM symptoms more frequently than those receiving tamoxifen therapy [26,37,38]. Furthermore, tamoxifen is associated with an increased likelihood of vaginal discharge [27,38].

Treatment options for GSM in BCSs

For alleviating GSM symptoms in postmenopausal women, the North American Menopause Society recommendations include non-hormonal moisturizers, vaginal lubricants, low-dose vaginal estrogen, vaginal dehydroepiandrosterone inserts, oral ospemifene, and systemic hormone therapy. However, due to the risk of breast cancer recurrence, estrogen-containing products might not be suitable for treating GSM in BCSs [3]. On the contrary, for BCSs who continue to experience genitourinary symptoms after non-hormonal therapies, the American College of Obstetricians and Gynecologists has suggested considering the use of low-dose vaginal estrogen, dehydroepiandrosterone, or testosterone supplementation [39]. The chemical compositions of non-hormonal vaginal gels or moisturizers vary widely, and their non-physiological pH, osmolarity, and additives can result in harmful effects, raising uncertainties about their suitability for prolonged treatment [40]. The heightened risk of cancer recurrence still hinders the prescription of vaginal hormone therapy [41,42]. A novel alternative, innovative vaginal laser therapy has been developed for such scenarios [6-8].

Types of laser treatment

The two most extensively researched lasers for alleviating GSM symptoms are the non-ablative erbium-doped yttrium aluminum garnet laser (Er:YAG) laser and microablative fractional carbon dioxide (CO_2_) laser [43]. These two lasers have different characteristics, such as the active medium, wavelength, and water absorption.

The non-ablative Er:YAG laser achieves deeper heating without cutting or overheating the mucosa. It emits light at 2,940 nm, which is highly absorbed in water [44,45]. The laser energy is rapidly absorbed in the top 5 μm of the epithelium, converting light energy into heat. There are two mechanisms: The first mechanism involves utilizing an optimal number of pulses (approximately four to seven) and an ideal long-pulse sequence duration (approximately 1-3 s). This approach maximizes the response of superficial tissues while keeping the exposure time short, resulting in a moderate coagulation depth. In the second mechanism, more pulses (typically 10-30) are delivered, along with an extremely extended pulse sequence (over 5 s), alongside local anesthesia. This combination allows for temperatures ranging from 65°C to 45°C at a 400-500 μm depth beneath the surface [46]. This approach is commonly called "smooth resurfacing" [47].

Moreover, the non-ablative Er:YAG laser comes together with a neodymium:YAG (Nd:YAG) laser; this configuration offers the advantage of simultaneous irradiation with both wavelengths [48]. The Nd:YAG laser can be directly irradiated onto the skin to efficiently deliver heat internally, as it is well absorbed by hemoglobin [49].

The fractional microablative CO_2_ laser uses a gas medium and emits pulses at a wavelength of 10,600 nm [43]. The absorption of this laser in human tissue is approximately 10 times lower than that of Er:YAG. In addition, the CO_2_ laser technology is not optimal for generating short laser pulses with high pulse energy. For these reasons, CO_2_ lasers are not suitable for non-ablative treatments with large laser-spot diameters. Instead, the CO_2_ laser is predominantly used in a fractional ablative mode [43] by drilling several small beam-diameter holes into the vaginal tissue.

The advent of modern laser treatments has challenged previously established theories. The Arrhenius model, an earlier theory, stated that the reaction rate increases exponentially with temperature. However, the mechanism of laser treatment should be characterized as a variable thermal shock response model, distinct from the standard Arrhenius model; this has been observed in investigations on non-ablative Er:YAG laser treatments, where a shift to higher critical temperatures for tissue damage than expected from the typical Arrhenius model has been observed, especially during extremely short exposure times. To explain this phenomenon, Lukač et al. introduced a novel model labeled the variable thermal shock response model, positing a synergistic influence of two biochemical processes that regulate cell survival and tissue damage during brief and prolonged exposure [50,51]. Lukač et al. discovered intense heat-shock biomodulation, where laser-generated heat prompts a cell response, releasing growth factors and boosting cell growth; intense heat-shock biomodulation utilizes Er:YAG lasers to deliver controlled intense heat shocks, aiding tissue regeneration while avoiding harm [51].

Histological changes in vaginal tissue after laser treatment

There is currently no histological investigation of laser treatment for BCSs. However, general information is available. According to the variable heat shock response model, Er:YAG laser and CO_2_ laser stimulate tissue remodeling and rejuvenation, promoting collagen neogenesis, formation of elastic fibers, and angiogenesis [52]. The Er:YAG laser's process leads to collagen remodeling and the initiation of neovascularization. These effects have been confirmed in histological studies.

According to a morphometric analysis of vaginal mucosal samples, Er:YAG laser irradiation increases the thickness of the epithelial layer and the number and volume density of capillaries. An analysis of vaginal biopsy samples did not show signs of inflammatory reactions, and no neutrophilic or eosinophilic infiltration was observed [53]. The average thickness of the epithelial layer before treatment was 45.0 μm, and after Er:YAG laser treatment, the average thickness of the epithelial layer increased to 152.9 μm [54]. In a pathological study of vulvodynia in patients with interstitial cystitis, the average thickness of the vaginal wall before treatment was reported to be 57.3 ± 14.2 μm, which increased to 133.3 ± 24.5 μm after Er:YAG laser treatment [55]. In a randomized controlled trial (RCT) of Er:YAG laser treatment involving 40 patients with lichen sclerosus, the thickness of sclerosis significantly decreased after laser treatment (-0.67 mm, 95% confidence interval: -0.99 to -0.34 mm, P = 0.009). By contrast, no significant change in thickness was observed after a treatment with corticosteroids in the control group (-0.10 mm, 95% confidence interval: -0.48 to 0.20 mm, P = 0.577) [56].

Microablative CO_2_ laser therapy aims at reconstructing the vaginal connective tissue. Electron microscopy examination has confirmed the presence of regenerating collagen fibers, highly expressed rough endoplasmic reticulum, and developed Golgi complexes [57]. These characteristics are associated with the stimulation of fibroblasts by generating collagen and other extracellular matrix components [57,58]. 

Efficacy of vaginal laser therapy in BCSs

Using local estrogen in BCSs is controversial, as conflicting evidence exists on whether vaginally administered estrogen increases serum estrogen levels and leads to a higher risk of breast cancer advancement or recurrence. Currently, there is no established answer concerning the safety of local estrogen treatment in patients with breast cancers, leading to a tendency to avoid therapy, which may negatively impact their quality of life [59].

Vaginal laser therapy has been evaluated in numerous studies of BCSs with VVA symptoms (Table 1) [6-8,60-75]. However, only one RCT is currently available [61]. In an RCT by Mension et al. involving 35 BCSs with VVA symptoms receiving AIs, vaginal CO_2_ laser treatment showed the same effectiveness as placebo treatment at the one-month follow-up [61]. Furthermore, several studies primarily employed single-arm prospective designs, and there were only four retrospective studies [7,60,71,72]. The sample sizes were relatively small, ranging from 16 [72] to 234 [60] patients.

**Table 1:** Vaginal laser research for BCSs BCS, breast cancer survivors; RCT, randomized controlled trial; FSFI, Female Sexual Function Index; FSDS-R, Female Sexual Distress Scale-Revised; SF-12, Short Form 12; PCS, Physical Component Summary; MCS12, Mental Component Summary 12; VAS, Visual Analog Scale; FSDI, Female Sexual Distress Index; GSM, genitourinary syndrome of menopause; ND:YAG, neodymium-doped yttrium-aluminum-garnet; PEG, Patient Global Impression of Change; PGI, Patient Global Impression; SPEQ, Short Personal Experiences Questionnaire; S-BIS, Spanish Body Image Scale; VEE, vaginal epithelium elasticity; VEL, vaginal erbium laser (SMOOTH mode); VET, vaginal epithelium thickness; VHIS, Vaginal Health Index Score; VMI, Vaginal Maturation Index; VVA, vulvovaginal atrophy

First author	Year	Country	Design	Laser type	Participants	Number of patients	Laser setting	Method	Assessed results	Subsequent monitoring	Outcome	Analysis of findings
Okui [60]	2023	Japan	Case-control study retrospective	VEL and Nd:YAG laser (VEL+Nd:YAG, Fotona)	BCS with GSM, vulvodynia, and dyspareunia	234	VEL: energy density 1.75 J/cm^2^, frequency 1.6 Hz, seven pulses every 5 mm along the vaginal canal, repeated three times. Nd:YAG: spot size 9 mm, energy density 90 J/cm^2^, pulse duration 5.0 s, six passes with brushing mode	One-month interval with three laser sessions	Symptoms assessed with VAS, FSFI, VHIS, and vulvodynia swab test	1, 3, 6, 12, and 24 months	Both lasers were effective in treating GSM pain.	VEL+Nd:YAG was more effective for superficial vulvar pain.
Mension [61]	2023	Spain	RCT	Monalisa Touch (SmartXide2 V2lr, DeKa), microablative fractional CO_2_ laser	BCSs undergoing treatment with aromatase inhibitors impacted by VVA	35 treated with vaginal laser; 37 treated with vaginal sham	SmartStack 2; double-pulse emission mode; dwell time 1000 μs; power 40 W; dwell time 1000 μs; spacing 1000 μm dot; delivery fluence 5.37 J/cm^2^	One-month interval with five laser sessions	VEE, VAS (dyspareunia), VHIS, VMI, FSFI, S-BIS, SF-12	1 month	VEE (↓), VAS (↓), VHI (↑), VMI (↑), FSFI (↑), S-BIS (↓). Demonstrated significant improvement without disparities between the groups.	After a six-month follow-up period, CO_2_ laser treatment was observed to be safe. However, there was no statistically significant difference compared to the placebo group.
Fidecicchi [62]	2022	Italy	Double-arm study, prospective	Hyperstack mode Er:YAG laser + VEL laser (Fotona)	Postmenopausal breast cancer survivors with GSM	68	VEL frequency 1.6 Hz, fluence 6.0 J/cm^2^. Frequency 1.6 Hz, releasing two stacks of 10 J/cm^2^, frequency of 1.6 Hz, fluence 1.5 J/cm, for each spot, 27 stacks	Three-month interval with three laser sessions	Superficial dyspareunia significantly improved in both groups; the Hyperstack group showed greater and persistent improvement.	3 months	Hyperstack treatment of the introitus and vestibulum in BCS leads to more significant improvement in superficial dyspareunia.	The VEL+HyperStack group resulted in more pronounced improvements compared to VEL alone.
Quick [63]	2021	USA	Single-arm study, prospective	Monalisa Touch (SmartXide2 V2lr, DeKa), microablative fractional CO_2_ laser	VVA in BCSs	59	SmartStack 1–3; dwell time 1000 µs; spacing 1000 µm; power 30 W	Interval of 30 to 45 days; a total of 3 laser sessions	FSDS-R, FSFI	12 months	FSDS-R, FSFI (↑). Significantly improved	In BCSs, CO_2_ laser treatment has been shown to enhance sexual function and alleviate VVA.
Salvatore [64]	2021	Italy	Single-arm study, prospective	Monalisa Touch (SmartXide2 V2lr, DeKa), microablative fractional CO_2_ laser	VVA in BCSs	40	SmartStack 1–3; spacing 1000 µm; dwell time 1000 µs; D-pulse; power 30 W	One-month interval with 5 laser sessions	VHIS, SF-12, FSFI, VAS, PCS, MCS12	1 month	VHI (↑), VAS (↓), FSFI (↑). Quality of life exhibited a significant improvement, with no discernible distinction between those receiving adjuvant therapy and those without it.	In BCSs, fractional CO_2_ laser treatment has been shown to enhance VVA and vaginal health. These improvements were observed without any variances linked to adjuvant therapy.
Siliquini [65]	2021	Italy	Single-arm study, prospective	Monalisa Touch (SmartXide2 V2lr, DeKa), microablative fractional CO_2_ laser	VVA in BCSs	45	SmartStack 1–3, spacing 1000 µm; dwell time 1000 µs; power 40 W	One-month interval with three laser sessions	VHIS, VAS, VVHI	3, 6, and 12 months	VHIS, VAS, and VVHI experienced a substantial and lasting improvement	In BCSs, fractional CO₂ vaginal laser treatment results in a durable enhancement of GSM symptoms, offering an effective and non-hormonal alternative.
Veron [66]	2021	France	Single-arm study, prospective	Monalisa Touch (SmartXide2 V2lr, DeKa), microablative fractional CO_2_ laser	VVA in BCSs	46	SmartStack 1–3, spacing 1000 µm; dwell time 1000 µs; power 26–40 W	One-month interval with three laser sessions	SF-12, FSFI	6 and 12 months	SF-12 (↓) and FSFI (↑). Over the past six months, the improvement in FSFI scores exhibited a reduction, yet the level of improvement remained higher compared to the initial baseline measurement.	In BCSs, CO_2_ laser treatment has shown efficacy in enhancing vaginal health and sexual function.
Hersant [67]	2020	France	Single-arm study, prospective	CO_2_ laser (iNtermedic’s Gynelasetm)	VVA in BCSs	20	Energy density or fluence 11.5 J/cm^2^; pulse width 0.9 ms	One-month interval with two laser sessions	VHIS, FSDI, VAS	6 months	VHI (↑), FSDI (↑), VAS (↓). Experienced a statistically significant improvement.	In BCSs, CO_2_ laser treatment has shown effectiveness in improving VVA and overall vaginal health.
Quick [68]	2020	USA	Single-arm study, prospective	Monalisa Touch (SmartXide2 V2lr, DeKa), microablative fractional CO_2_ laser	VVA in BCSs	64	SmartStack 1–3, spacing 1000 µm; dwell time 1000 µs; power 30 W	30–45-day interval with three to four laser sessions	Vaginal pH, PGI, VAS, FSFI	At the end of the treatment	VAS (↓), FSFI (↑). There was an improvement in the quality of life. Enhanced vaginal pH exhibited a reduction.	CO_2_ laser treatment has been shown to enhance vaginal health, sexual function, and alleviate VVA in BCSs.
Areas [69]	2019	Brazil	Single-arm study, prospective	Etherea-mX er:YAG laser (Vydance, Brazil)	VVA in BCSs	24	Frequency 0.5 Hz, fluency 2.0 J/cm^2^; smooth mode	One-month interval with three laser sessions	Sexual function, VHIS, SPEQ	1 month	Sexual function score (↑), VHIS (↑). SPEQ significantly improved.	Er:YAG laser treatment exhibited efficacy in enhancing vaginal health and sexual function among BCSs.
Pearson [70]	2019	Australia	Single-arm study, prospective	Monalisa Touch (SmartXide2 V2lr, DeKa), microablative fractional CO_2_ laser	VVA in BCSs	25	SmartStack 1–3, spacing 1000 µm; dwell time 1000 µs; power 30 W	One-month interval with three laser sessions	FSFI, SF-12, VAS, Likert scale	1 month	FSFI (↑), VAS (↓) Experienced significant improvement. Quality of life showed enhancement. satisfaction with the treatment.	CO_2_ laser treatment has shown efficacy in enhancing VVA, sexual function, and overall quality of life in BCSs.
Mothes [71]	2018	Germany	Single-arm study, retrospective	MCl 31 (Asclepion, Germany) Dermablate dual-phase protocol pulsed ablative Er:YAG laser	VVA in BCSs	16	First phase: 300-µs pulse duration, 15–35 J/cm^2^ fluence, 0.5–2-s pulse interval. Second phase: 1000-µs pulse duration, 3–9 J/cm^2^ fluence, 0.5–2-s pulse interval	One session	VHIS, vaginal pH	6 weeks	VHIS (↑). No statistically significant decrease in vaginal pH.	Er:YAG laser treatment has shown effectiveness in enhancing vaginal health among BCSs
Pagano [72]	2018	Italy	Single-arm study, retrospective	Monalisa Touch (SmartXide2 V2lr, DeKa), microablative fractional CO_2_ laser	VVA in BCSs	82	SmartStack 1–3, spacing 1000 µm; dwell time 1000 µs; power 30 W	One-month interval with three laser sessions	VAS	1 month	VAS (↓)	CO_2_ laser treatment has been shown to improve VVA in BCSs.
Becorpi [73]	2017	Italy	Single-arm study, prospective	Monalisa Touch (SmartXide2 V2lr, DeKa), microablative fractional CO_2_ laser	VVA in BCSs	20	SmartStack 1–3, spacing 1000 µm; dwell time 1000 µs; power 30 W	One-month interval with two laser sessions	VHIS, FSFI, VAS	1 month	VHI (↑), FSFI (↑), VAS (↓)	CO_2_ laser treatment has demonstrated improvement in vaginal health, sexual function, and VVA in BCSs.
Gambacciani [8]	2017	Italy	Double-arm study, prospective	VEL laser (Fotona)	VVA in BCSs	37	Fluence 6.0 J/cm^2^, frequency 1.6 Hz	One-month interval with three laser sessions	VHIS, VAS	1, 3, 6, 12, and 18 months	VHIS (↑) and VAS (↓) at 12 months. No statistically significant difference observed at 18 months.	Er:YAG laser treatment has been shown to enhance vaginal health and alleviate VVA in BCSs.
Pagano [74]	2016	Italy	Single-arm study, retrospective	Monalisa Touch (SmartXide2 V2lr, DeKa), microablative fractional CO_2_ laser	VVA in BCSs	26	SmartStack 1–3, spacing 1000 µm; dwell time 1000 µs; power 30 W	One-month interval with three laser sessions	VHIS, VAS	VHIS: 4 weeks; VAS: 11 months	VHIS (↑) and VAS (↓). Experienced a significant improvement (with no distinctions between the aromatase inhibitor group, tamoxifen, and no adjuvant therapy group).	CO_2_ laser treatment has been shown to enhance vaginal health and alleviate VVA in BCSs, with no variations related to adjuvant therapy.
Pieralli [75]	2016	Italy	Single-arm study, prospective	Monalisa Touch (SmartXide2 V2lr, DeKa), microablative fractional CO_2_ laser	VVA in BCSs	50	SmartStack 1–3, spacing 1000 µm; dwell time 1000 µs; power 30 W	One-month interval with three laser sessions	VHIS, VAS	11 months	VHIS (↑) and VAS (↓)	CO_2_ laser treatment has been demonstrated to enhance vaginal health and alleviate VVA in BCSs.
Bojanini [7]	2016	Colombia	Single-arm study, prospective	VEL laser (Fotona)	VVA in BCSs	40	RenovaLase: 2940nm Er:YAG laser, non-ablative mode	Three-week interval with two laser sessions	Reduction of vaginal dryness, dyspareunia, and intercourse avoidance	12 months	Statistically significant improvement	Laser treatment is effective and safe for reducing GSM symptoms in both natural and therapy-induced menopause patients.
Bojanini [6]	2014	Colombia	Interventional	VEL laser (Fotona)	Women with natural menopause after post-gynecological cancer	20 (Group A), 20 (Group B)	RenovaLase: 2940nm Er:YAG laser, non-ablative mode	Three-week interval with two laser sessions	Vaginal dryness, dyspareunia, frequency, satisfaction	3 months	Statistically significant improvement	The study demonstrated the efficacy and safety of laser treatment for vaginal atrophy in all groups of women. Transient adverse effects were minimal.

Various settings using Er:YAG and CO_2_ lasers have been used in BCSs. Research protocols involve laser sessions ranging from three to five times, with four- to eight-week intervals. In many studies, three laser treatments are commonly used. Recently, laser irradiation for the vagina and the entire external genitalia has been suggested to be more effective. Er:YAG and CO_2_ lasers have been reported to improve vaginal health. Improvement in VVA symptoms was evaluated using a 0-10 VAS. The treatment resulted in the recovery of vaginal atrophy and significantly improved the quality of life. Studies assessing sexual function through FSFI or Female Sexual Distress Scale (FSDS) scores have confirmed improvements in vaginal health status. These studies conducted extended follow-up periods of 24 [60] and 18 months [8]. The investigation periods in many studies ranged from 1 to 12 months.

Four studies [8,25,65,66] evaluated the long-term sustainability of GSM symptom improvement in BCSs, and the results were promising. Okui et al. [60] also reported efficacy at 24 months in a retrospective study. Quick et al. [64] highlighted a potential long-term benefit of laser therapy by noting that sexual function was improved even two years after treatment completion. Veron et al. [67] demonstrated sustained improvement in urinary and sexual function through an 18-month follow-up study. However, there was a tendency for the effects to decrease at six months after the treatment. Siliquini et al. [66] reported improvements up to 12 months after the treatment completion in BCSs. Gambacciani et al. [8] indicated a duration of 12-18 months for the persistence of the effects of laser treatment.

Salvatore et al. [64] reported a study involving 40 BCSs that evaluated the efficacy of microablative fractional CO_2_ laser therapy for VVA symptoms. The participants were divided into two groups based on their hormone therapy status. The study found no significant differences in VVA symptoms, vaginal health, and sexual function between the two groups, suggesting that adjuvant therapy does not affect treatment outcomes, irrespective of the hormone therapy status. Pagano et al. [72] reported that the effectiveness of CO_2_ laser therapy was significant in 82 BCSs, as confirmed by a multivariate analysis, regardless of patients' age and type of adjuvant therapy.

The studies by Okui et al. [60] and Fidecicchi et al. [62] demonstrated that expanding laser therapy to not only the vagina but also the external genitalia improved treatment efficacy in BCSs; this suggests that the cause of GSM in BCSs is not limited to the vagina. Cucinella et al. [76] concluded from reviewed studies that vaginal erbium laser and local hyaluronic acid are safe options for addressing genitourinary symptoms in BCSs. Both treatments demonstrated improvements in urogenital health and quality of life.

Safety of vaginal laser procedures

The U.S. Food and Drug Administration (FDA) issued a warning regarding using energy-based devices in 2018; this included lasers and radiofrequency for treating genital disorders related to menopause, vaginal rejuvenation, and vaginal cosmetic procedures [77]. The American Urogynecologic Society has determined the need to provide a clinical consensus statement applicable in situations where there is limited evidence regarding the use of vaginal energy-based devices and convened a panel of experts to compile the most important agreed-upon expert opinions [78]. Guo et al. conducted a systematic review of articles published before September 2019, revealing three publications that detailed 29 presumed laser-related complications, of which only five (17.2%) did not report worsened symptoms after treatment [79]. Guo et al. suggested that the FDA's safety communication lacks substantiation and calls for additional trials [79]. Gambacciani et al. collected information on the frequency of adverse events associated with non-ablative Er:YAG laser treatment from 113,174 patients. They shared detailed information from 62,727 patients and provided information on the frequency of observed adverse events in a population of 43,095 patients. They reported that all observed adverse events were mild to moderate in severity, transient, and occurred at a low frequency [80]. Gambacciani and Cervigni addressed the FDA's warning, highlighting the lack of approval for fractional ablative CO_2_ lasers in the treatment of stress urinary incontinence and GSM, as well as the potential association with serious adverse events [81]. Furthermore, they reported on the treatment effectiveness and safety of non-ablative Er:YAG laser [80,81].

All evaluated studies reported no serious adverse events, ensuring consistency in the research (Table 2). In three long-term observational studies, no serious adverse events were observed [8,60,63]. In the RCT, the vaginal laser treatment group exhibited no significant increase in adverse events, compared with the control group [61]. Some patients reported discomfort and pain during probe insertion [60,61,63,66], but these side effects were mild, and patients recovered within a few days. Multiple studies have reported reduced discomfort associated with vaginal procedures after three laser sessions. Two cases of vaginal candidiasis and acute cystitis were reported [69].

**Table 2:** Adverse events in the evaluated studies

First author	Year	Adverse events in the evaluated studies
Okui [60]	2023	No superficial surgical site infections (SSIs) were observed. The most frequent complaints among the participants were transient and moderate heating sensations during treatment, vaginal discharge, mild edema, dryness, transient de novo urinary incontinence, and itching. All minor complications lasted only a few days after treatment, and no participant complained of adverse events lasting longer than one year.
Mension [61]	2023	Mild adverse events (e.g., spotting or vaginal itching) were experienced by approximately 45% of the patients during five treatments. Moderate complications (including urinary tract infections) were observed in approximately 10% of the patients. No severe adverse events were reported.
Fidecicchi [62]	2023	No significant adverse events were recorded. Some women reported temporary warm sensations during treatment, not described as burning. In a few cases, self-limiting leukorrhea was reported for a few days post treatment.
Quick [63]	2021	No serious adverse events were reported.
Salvatore [64]	2021	Mild adverse events (e.g., inflammation of the external genitalia) resolved naturally. No severe adverse events were observed.
Siliquini [65]	2021	No serious adverse events were reported.
Veron [66]	2021	The study states that no serious adverse effect directly linked to laser therapy was recorded during the study period. The main minor adverse effects were discomfort grade 1 and small vaginal bleeding during the day after laser therapy.
Hersant [67]	2020	No serious adverse events were reported.
Quick [68]	2020	Vaginal discharge and vaginal dryness
Arêas [69]	2019	Vaginal candidiasis (one woman); acute cystitis (one woman)
Pearson [70]	2019	No serious adverse events were reported.
Mothes [71]	2018	No serious adverse events were reported.
Pagano [72]	2018	No serious adverse events were reported.
Becorpi [73]	2018	No serious adverse events were reported.
Gambacciani [74]	2017	No serious adverse events were reported.
Pieralli [8]	2016	No serious adverse events were reported.
Pagano [7]	2016	No serious adverse events were reported.
Bojanini [6]	2016	No serious adverse events were reported.

In addition, three women had abnormal Pap smears (two with low-grade and one with high-grade squamous intraepithelial lesions). The relationship between vaginal laser application and human papillomavirus infection remains unclear [66]. Some patients experienced pain, burning, and itching during the 6-, 9-, and 12-month follow-ups, likely due to the treatment's effect diminishing and GSM reappearing [6]. Furthermore, no complications related to the study procedure were reported, even during follow-ups of over 12 months. Numerous studies on safety have indicated that vaginal laser treatment with appropriate parameter settings does not lead to adverse events. Under these conditions, it is believed that symptoms associated with GSM can be safely addressed.

Limitations

Several concerns have been raised regarding the quality of evidence supporting vaginal laser treatment for GSM [82]. Many unresolved issues exist around the optimal placement of vaginal laser therapy within clinical practice. They include concerns about treatment effectiveness, cost-effectiveness, long-term safety, and the regulatory framework governing its utilization [82]. Standardization is needed in terms of laser settings, research procedures, and reported outcomes to improve the reproducibility of laser studies. While vaginal laser therapy may hold promise in alleviating the symptoms of GSM, there is an opinion that it should be used cautiously and primarily as a part of research until more robust evidence is available [83].

These concerns extend beyond BCSs and focus broadly on vaginal laser therapy for GSM. Several RCTs on vaginal laser treatment have reported varying results. For instance, Salvatore et al. [84] and Ruanphoo and Bunyavejchevin [85] demonstrated a significantly higher efficacy of microablative fractional CO_2_ laser treatment than that of placebo in their respective trials. However, O'Reilly et al., in an RCT using non-ablative Er:YAG laser therapy, showed significant improvements compared to a placebo [86]. By contrast, RCTs utilizing fractional microablative CO_2_ laser therapy by Page et al. [87] and Mension et al. [61] did not confirm the superiority of laser treatment for GSM.

Regarding the methods employed in the studies evaluated in this review, there is reported heterogeneity in laser settings, both in prospective/retrospective single-arm studies and RCTs. This variability in laser parameters results in differing energy levels delivered to the vaginal surface, potentially influencing tissue bioactivation. Differences in the power levels and total energy delivered and the mode of delivery (e.g., microablative or non-ablative effect, fractional administration, and depth of penetration) contribute to variations in vaginal tissue modifications. Therefore, it is crucial to continually explore effective approaches, as seen in studies, such as Okui et al.'s combination of two types of lasers [48] and Fidecicchi et al.'s expansion of the irradiation area [68]. These factors contribute to the challenges of conducting large-scale, multi-center RCTs with standardized settings over extended periods.

## Conclusions

The importance of researching alternative treatments for GSM symptoms is evident, given the increasing incidence of breast cancer among women, some of whom may experience refractory GSM. Among the proposed therapies, thermal energy treatments, such as vaginal laser therapy, have shown effectiveness and safety in treating VVA.

Although only one RCT exist with BCSs, numerous prospective and retrospective studies have demonstrated vaginal laser therapy's positive impact on VVA symptoms, vaginal health, sexual function, and overall quality of life. Further research is needed to establish its long-term efficacy and safety, emphasizing standardized and controlled investigations.

In conclusion, vaginal laser therapy holds potential as a valuable approach for improving the quality of life in BCSs experiencing genitourinary symptoms related to menopause and breast cancer treatments.
